# A natural *Anopheles*-associated *Penicillium chrysogenum* enhances mosquito susceptibility to *Plasmodium* infection

**DOI:** 10.1038/srep34084

**Published:** 2016-09-28

**Authors:** Yesseinia I. Angleró-Rodríguez, Benjamin J. Blumberg, Yuemei Dong, Simone L. Sandiford, Andrew Pike, April M. Clayton, George Dimopoulos

**Affiliations:** 1 W. Harry Feinstone Department of Molecular Microbiology and Immunology, Johns Hopkins Bloomberg School of Public Health, 615 N Wolfe St., Baltimore, MD 21205, USA.

## Abstract

Whereas studies have extensively examined the ability of bacteria to influence *Plasmodium* infection in the mosquito, the tripartite interactions between non-entomopathogenic fungi, mosquitoes, and *Plasmodium* parasites remain largely uncharacterized. Here we report the isolation of a common mosquito-associated ascomycete fungus, *Penicillium chrysogenum*, from the midgut of field-caught *Anopheles* mosquitoes. Although the presence of *Pe. chrysogenum* in the *Anopheles gambiae* midgut does not affect mosquito survival, it renders the mosquito significantly more susceptible to *Plasmodium* infection through a secreted heat-stable factor. We further provide evidence that the mechanism of the fungus-mediated modulation of mosquito susceptibility to *Plasmodium* involves an upregulation of the insect’s ornithine decarboxylase gene, which sequesters arginine for polyamine biosynthesis. Arginine plays an important role in the mosquito’s anti-*Plasmodium* defense as a substrate of nitric oxide production, and its availability therefore has a direct impact on the mosquito’s susceptibility to the parasite. While this type of immunomodulatory mechanism has already been demonstrated in other host-pathogen interaction systems, this is the first report of a mosquito-associated fungus that can suppress the mosquito’s innate immune system in a way that would favor *Plasmodium* infection and possibly malaria transmission.

Several studies have demonstrated a profound influence of the *Anopheles gambiae* midgut bacterial microbiota on *Plasmodium* infection of the mosquito. This influence has been attributed to a bacteria-mediated stimulation of anti-*Plasmodium* immune defenses and the production of anti-*Plasmodium* metabolites^1^,^2^,^3^. In comparison to the bacterial microbiota, much less is known about the mosquito-associated fungi, or mycobiome, and how these organisms may influence infection with, and transmission of, disease-causing pathogens such as *Plasmodium*. Most studies of mosquito-associated fungi have focused on the entomopathogenic fungi because of their potential for mosquito control^4^,^5^. Filamentous fungi and yeasts have been isolated from mosquito midguts and other tissues, but their influence on mosquito-*Plasmodium* interaction has not been extensively addressed^6^,^7^,^8^,^9^. A recent study showed that a *Anopheles*-associated yeast strain produced an antiparasitic toxin^10^. Fungi produce an extensive range of proteins and secondary metabolites, making them attractive sources for the discovery of novel bioactive molecules with desirable properties^11^,^12^. Here we report the isolation of a commonly mosquito-associated ascomycete fungus, *Penicillium chrysogenum*, from the midgut of field-collected *Anopheles* mosquitoes. We show that the presence of *Pe. chrysogenum* in the mosquito midgut does not influence mosquito longevity but instead its susceptibility to the malaria parasite *Plasmodium*. We further show that the molecular basis of this phenomenon likely involves fungus-mediated upregulation of ornithine decarboxylase (ODC), sequestering L-arginine for polyamine biosynthesis and thereby resulting in diminished production of anti-parasitic nitric oxide (NO).

## Results

A *Penicillium chrysogenum fungus* was isolated from the midgut of *Anopheles* mosquitoes collected in the Maunabo region of Puerto Rico. Visual inspection of conidial structure by light microscopy, in combination with DNA sequencing of the ribosomal internal transcribed spacer (ITS) region, identified this fungus as a strain of *Penicillium chrysogenum*^13^. *Pe. chrysogenum* is found globally in temperate and subtropical regions, and it has been isolated from various mosquito species, including malaria vectors^8^,^9^. In fact, over 50% of fungi infecting field-caught *Anopheles* have been identified as *Penicillium*^8^. These results suggest that *Pe. chrysogenum* may be an opportunistic mosquito-associated fungus. *Pe. chrysogenum* is generally considered non-pathogenic, although some of its strains are capable of producing harmful mycotoxins^14^. Since the fungus was isolated from the mosquito’s midgut tissue, it is likely that mosquitoes are acquiring *Pe. chrysogenum* in nature by feeding on contaminated sugar and water sources. Thus, in the present study we investigated the influence of orally administered *Pe. chrysogenum* on *A. gambiae* in the context of mosquito longevity as well as susceptibility to *Plasmodium* infection and the midgut microbiota.

### *Pe. chrysogenum* does not affect *A. gambiae* survival

**Figure 1:**
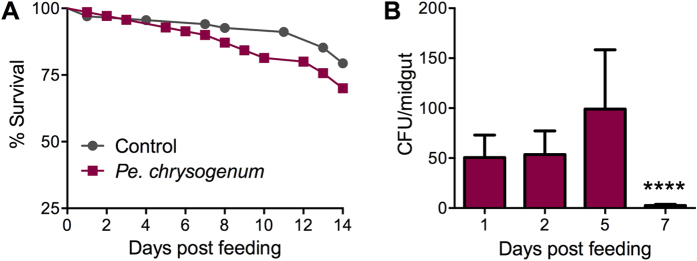
Impact of *Pe. chrysogenum* on *A. gambiae* longevity and fungus colonization efficiency of the mosquito midgut. (**A**) Survival of adult female *A. gambiae*, over 14 days, after feeding on a *Pe. chrysogenum*-laced sucrose solution for 48 h. Data represent 4 biological replicates (N = 79, *P* = 0.180), Kaplan-Meier statistical analysis. (**B**) *Pe. chrysogenum* colony-forming units (CFU) per midgut at 1, 2, 5, and 7 days after ingestion of fungus via a sucrose solution for 48 h (N = 40 at day 1, 5, 7, and N = 39 at day 2). CFU for *Pe. chrysogenum* were significantly lower (*P* = 0.0001) on day 7 than on day 1. Three biological replicates were performed; bars represent standard error of the mean (SEM). ****P ≤ 0.0001; two-tailed Mann-Whitney test.

To gain baseline information as to whether the presence of *Pe. chrysogenum* in the mosquito midgut could influence mosquito physiology in either an adverse or beneficial manner, we investigated its impact on mosquito longevity as a standard fitness parameter. We allowed adult female *A. gambiae* to sugar-feed on sucrose containing *Pe. chrysogenum* conidia (2.5 × 10^8^ conidia/mL) for 48 h and then monitored mosquito mortality over a 14-day period. Mosquitoes fed on *Pe. chrysogenum*-laced sucrose experienced the same mortality level as did the naïve sucrose-fed controls (Fig. 1A). In general, *Penicillium* fungi are considered non-pathogenic, and this result indicates that *Pe. chrysogenum* is not exerting a major detrimental effect on *A. gambiae*, corroborating a previous report showing low overall mosquito mortality after exposure to conidia at a concentration four times higher than the one we used^15^. To investigate the midgut colonization capacity of the fungus, we assayed fungal colony forming units recovered from *A. gambiae* midguts over the course of the 7 days immediately following feeding on a fungus-laced sucrose solution (Fig. 1B). *Pe. chrysogenum* reached its highest infection intensity by day 5 and dropped to a significantly lower level at day 7 (*P* = 0.0001 when compared to day 1).

### Midgut colonization of *Pe. chrysogenum* enhances mosquito susceptibility to *Plasmodium* infection

**Figure 2:**
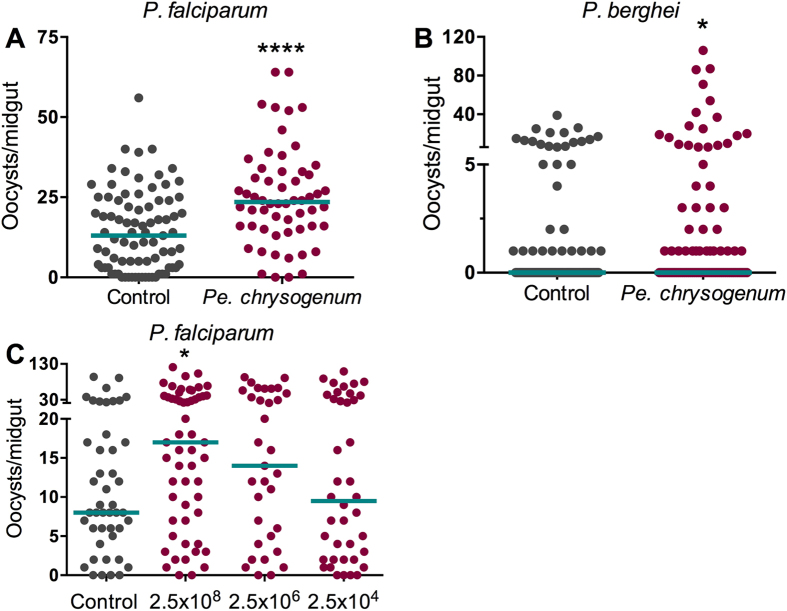
Influence of *Pe. chrysogenum on Plasmodium* infection. (**A**) Influence of *Pe. chrysogenum* on *P. falciparum* infection of *A. gambiae*, as a measured by oocyst numbers 7 days after feeding on a *P. falciparum* gametocyte culture (infection intensity). The mosquito cohort (N = 58) that had been exposed to a *Pe. chrysogenum*-laced sucrose solution for 48 h prior to parasite infection had a significantly higher *P. falciparum* infection (*P* = 0.0001) than did the non-fungus-exposed control cohort (N = 83). (**B**) Influence of *Pe. chrysogenum* on *P. berghei* infection intensity in *A. gambiae*. The mosquito cohort (N = 89) that had been exposed to a *Pe. chrysogenum*-laced sucrose solution for 48 h prior to parasite infection had a significantly higher infection (*P* = 0.0161) than did the non-fungus-exposed control cohort (N = 104). (**C**) Influence of various doses of *Pe. chrysogenum* on *P. falciparum* infection intensity. Mosquito cohorts that had been exposed to *Pe. chrysogenum* at 2.5 × 10^8^ conidia/mL (N = 55, *P* = 0.0137); 2.5 × 10^6^ conidia/mL (N = 33, *P* = 0.1449), or 2.5 × 10^4^ conidia/mL (N = 40, *P* = 0.1866) for 48 h prior to parasite infection had a significantly higher level of *P. falciparum* infection than did the non-fungus-exposed control cohort (N = 45). Graphs show three independent biological replicates. Each dot represents a single midgut, and bars represent the median. *P < 0.05, ****P ≤ 0.0001; two-tailed Mann-Whitney test.

To investigate whether the presence of *Pe. chrysogenum* in the mosquito midgut could influence *Plasmodium* infection, we fed adult female *A. gambiae* on a conidia-laced sucrose solution (2.5 × 10^8^ conidia/mL) for 48 h before providing the mosquitoes with an infectious blood meal containing *P. falciparum* gametocytes. The presence of *Pe. chrysogenum* in the mosquito midgut resulted in an enhanced (*P* = 0.0001) *P. falciparum* infection when compared to naïve sugar-fed controls (Fig. 2A). To investigate whether the *Pe. chrysogenum*-mediated influence on parasite infection was *Plasmodium* species-specific, we performed a similar assay with the rodent malaria parasite *P. berghei*. The presence of *Pe. chrysogenum* in the mosquito midgut again resulted in a higher (*P* = 0.0161) *P. berghei* infection (Fig. 2B) when compared to controls, suggesting that the infection enhancement is parasite species-independent^16^. In nature, mosquitoes will consume sugar and water from a variety of sources, providing opportunities for fungi and other microorganisms to enter the midgut lumen^17^,^18^. It is impossible to exactly reproduce this fashion of fungus acquisition in the laboratory, since the concentrations of *Pe. chrysogenum* in these different natural habitats and media are unknown. However, to investigate whether the *Pe. chrysogenum*–dependent modulation of parasite infection is dose-dependent, we performed assays providing various concentrations of the fungus in the sucrose solution prior to *P. falciparum* infection. We observed a dose-dependent increase in *P. falciparum* infection using live fungi (Fig. 2C). These results suggest that both the dose of fungi and variance in individual mosquito susceptibility to fungal colonization play a role in determining whether *Pe. chrysogenum* will modulate *Plasmodium* susceptibility.

### *Pe. chrysogenum* modulates *Plasmodium* infection through a secreted heat-stable factor

**Figure 3:**
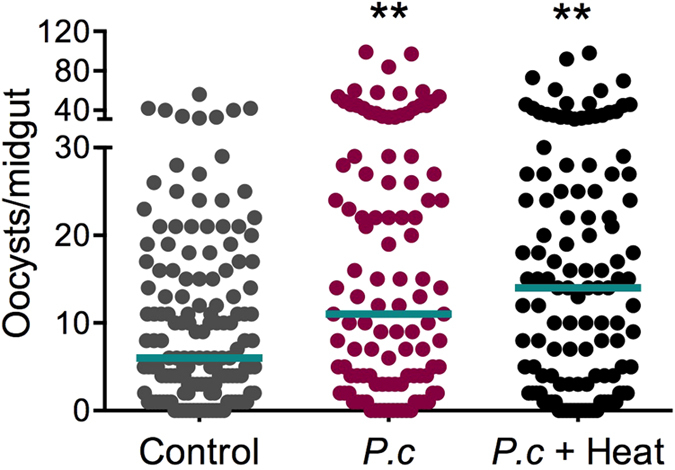
Influence of *Pe. chrysogenum* culture filtrate on *Plasmodium* infection. Measured by oocyst numbers at 7 days after feeding on a *P. falciparum* gametocyte culture, the mosquito cohort (N = 109) that had been exposed to a fungus culture filtrate-laced sucrose solution and the cohort (N = 120) that had been exposed to a heat-treated fungus culture filtrate-laced sucrose solution for 48 h prior to parasite infection had significantly higher infection levels (*P* = 0.0067 for filtrate and *P* = 0.0018 for heat-treated filtrate) when compared to the non-fungus-exposed control cohort (N = 143). Graph represent three independent biological replicates, each dot represents a single midgut, and bars represent the median. **P < 0.01; two-tailed Mann-Whitney test.

Fungi typically produce a variety of proteins and secondary metabolites^17^,^18^,^19^. To investigate whether *Pe. chrysogenum*’s influence on *Plasmodium* infection could be attributed to a secreted factor, we fed mosquitoes on a sucrose solution containing a *Pe. chrysogenum* culture filtrate (devoid of fungi) for 48 h prior to *P. falciparum* infection. Feeding on the fungus filtrate resulted in a significant increase in *P. falciparum* infection intensity (*P* = 0.0067) when compared to sterile sucrose-fed controls (Fig. 3A). This result suggests that *Pe. chrysogenum* influences parasite infection of the mosquito midgut through a secreted factor. We next investigated whether this secreted factor was heat-stable. We heat-treated the fungus filtrate at 95 ^o^C for 2 h before providing it to mosquitoes and infecting them with *P. falciparum*. Surprisingly, we again observed a significant increase in the infection intensity (*P* = 0.0018) of *P. falciparum* infection when compared to the sterile sucrose-fed controls (Fig. 3A). These findings suggest that *Pe. chrysogenum* produces a heat-stable, secreted secondary metabolite, or peptide, that enhances *Plasmodium* infection in the mosquito.

### *Pe. chrysogenum* does not affect *Plasmodium* development in a mosquito-independent fashion

**Figure 4:**
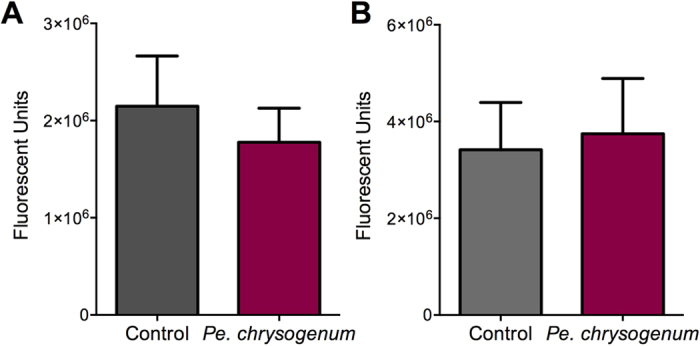
Influence of *Pe. chrysogenum* on *in vitro Plasmodium* development and ookinete viability. (**A**) No difference in *in vitro P. berghei* ookinete development or viability was observed between *Pe. chrysogenum* conidia-supplemented ookinete cultures (N = 18, *P* = 0.5521) and non-*Pe. chrysogenum*-exposed control cultures (N = 9). (**B**) No difference in *in vitro P. berghei* ookinete development or viability was observed between *Pe. chrysogenum* culture filtrate-supplemented cultures (N = 15, *P* = 0.8321) and non-supplemented control cultures (N = 12). Values are arbitrary fluorescence; three biological replicates were performed, bars represent standard error of the mean (SEM), two-tailed unpaired t-test.

Next, we investigated whether the effect of *Pe. chrysogenum* on *Plasmodium* infection could be attributed to a direct interaction between the fungus-secreted molecule(s) and the parasite, operating in a mosquito-independent fashion. We exposed an *in vitro* ookinete culture of a luciferase–expressing *P. berghei* parasite to the *Pe. chrysogenum* conidia (*P* = 0.5521) or filtrate (*P* = 0.8321) and monitored ookinete-stage parasite development and viability. The presence of the *Pe. chrysogenum* or fungus-secreted molecule(s) did not influence parasite development or viability when compared to the non–exposed control parasite culture (Fig. 4A,B). These results suggest that the effect of *Pe. chrysogenum* on *Plasmodium* infection is likely mediated through a modulation of mosquito susceptibility rather than parasite development.

### The mosquito microbiota is not influencing the fungus-mediated modulation of mosquito susceptibility to *Plasmodium* infection

**Figure 5:**
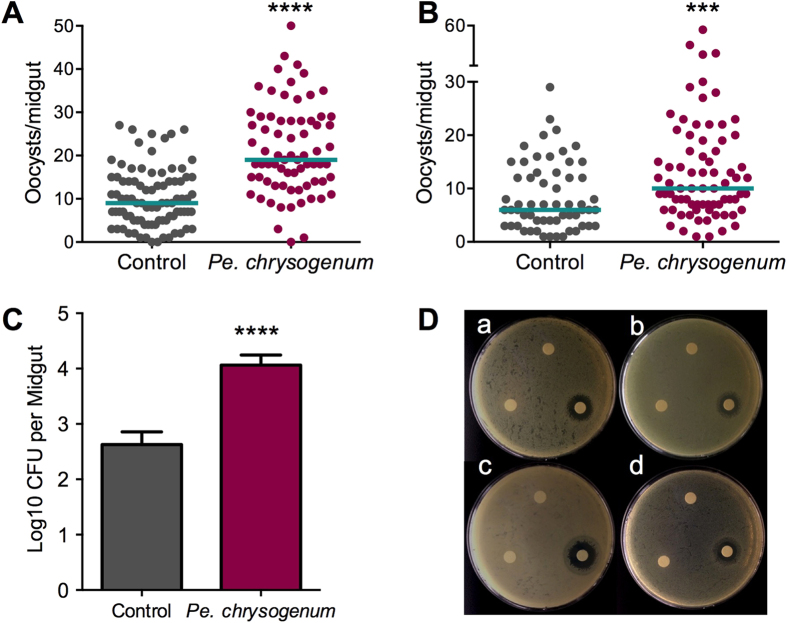
The influence of *Pe. chrysogenum* on *Plasmodium* infection is microbiota-independent. *P. falciparum* infection intensity, as measured by oocyst numbers at 7 days after feeding on a gametocyte culture, was significantly higher in (**A**) *Pe. chrysogenum conidia*-exposed mosquitoes (N = 72, *P* = 0.0001) and (**B**) fungus culture filtrate-exposed (N = 75, *P* = 0.0009) mosquitoes than in their respective bacteria-void, antibiotic-treated non-*Pe. chrysogenum*-exposed controls (N = 87, N = 59, respectively). Graphs show three independent biological replicates; each dot represents a single midgut, and horizontal bars represent the median infection intensity. ***P < 0.001, ****P < 0.0001; two-tailed Mann-Whitney test. (**C**) Midgut bacterial load, as measured by colony forming units (CFU), was significantly higher in *Pe. chrysogenum*-exposed mosquitoes (*P* = 0.0001) than in non-exposed controls (N = 25 for each). Error bars represent the standard error of the mean. ****P ≤ 0.0001; two-tailed Mann-Whitney test. (**D**) *Pe. chrysogenum* does not produce anti-bacterial products. Image shows four mosquito-isolated bacteria plates: (a) *S. marcences*, (b) *E. hormaechei*, (c) *B. subtilis*, and (d) *S. capprae* grown with a disk soaked in a fungus culture filtrate-laced sucrose solution (left disc) or a fungus culture filtrate-laced water solution (top disc), as compared to an antibiotic-soaked disc (right disc).

*Penicillium* fungi are known to produce antibiotics, and our previous studies have shown that antibiotic-mediated suppression of the midgut microbiota enhances *Plasmodium* infection in *Anopheles*^1^,^22^. To investigate whether the mosquito-associated bacteria could in some way be involved in the fungus-mediated modulation of *Plasmodium* infection, we suppressed the *A. gambiae* midgut microbiota by maintaining the mosquitoes on a sucrose solution containing a broad-spectrum antibiotic cocktail, prior to ingestion of the fungus filtrate and subsequent infection with *P. falciparum*. Ingestion of the *Pe. chrysogenum* conidia (*P* = 0.0001) or filtrate (*P* = 0.0009) still caused a significant increase in *P. falciparum* infection of antibiotic-treated mosquitoes when compared to the sterile sucrose-fed controls (Fig. 5A,B). These results suggest a bacteria-independent mechanism underlying the observed increase in *P. falciparum* infection after *Pe. chrysogenum* exposure.

We also investigated the impact of *Pe. chrysogenum* exposure on the midgut bacterial load by feeding *A. gambiae* on conidia-laced sucrose solution for 2 days and subsequently enumerating the bacteria using colony forming unit (CFU) assays. Interestingly, the presence of *Pe. chrysogenum* in the mosquito midgut resulted in a significant increase (*P* = 0.0001) in the midgut bacterial load (Fig. 5C). We also found that *Pe. chrysogenum* did not produce any antibiotic compound with antibacterial activity (Fig. 5D). *Pe. chrysogenum*’s impact on both parasitic and bacterial infection suggests that it involves the suppression of a physiological system of the mosquito that can act against both bacteria and the parasite, such as the innate immune system^1^,^2^,^3^,^4^,^5^,^6^,^7^,^8^,^9^,^10^,^11^,^12^,^13^,^14^,^15^,^16^,^17^,^18^,^19^,^20^,^21^,^22^,^23^. Alternatively, the fungus-produced factor could enhance a mosquito physiological system that favors both parasitic and bacterial infection.

### A *Pe. chrysogenum* filtrate induces *A. gambiae* genes that influence *Plasmodium* infection

The influence of *Pe. chrysogenum* on both *Plasmodium* infection and the midgut microbiota as well as the lack of influence on the *in vitro* development and viability of ookinete-stage parasites strongly implied the involvement of a physiological system in the mosquito. To gain insight into how the fungus might influence mosquito physiology at the molecular level, we compared the transcriptomes of mosquitoes that had been provided with heat-inactivated fungus filtrate through a sucrose solution for 48 h to a control cohort maintained on a sterile sucrose solution. The experiment showed an overall regulation of a relatively small number of genes that included 32 induced genes belonging to the following functional groups: metabolism (16%), immunity (16%), redox/stress (13%), digestion (13%), cytoskeletal and structural (3%), transport (3%), diverse functions (20%), and unknown function (16%) (Table 1). The gene showing the greatest increase (2.94-fold) in mRNA abundance upon exposure to *Pe. chrysogenum* filtrate encoded an ornithine decarboxylase (ODC). ODC is an enzyme that mediates polyamine synthesis, using L-arginine (L-Arg) as a substrate.

Because OCD has been shown to modulate infection by pathogens, including *Plasmodium* infection of mosquitoes^24^,^25^, we hypothesized that it was likely to be responsible for mediating the effect of *Pe. chrysogenum* on *Plasmodium* infection. Chemical inactivation of OCD by feeding mosquitoes on the inhibitor a-difluoromethylornithine (DFMO) via sucrose solution was previously shown to result in a greater resistance to *P. berghei* infection^26^. Another study has shown that *Chlamydia trachomatis* modulates the immune competence of macrophages by upregulating OCD, which in turn results in L-Arg sequestration, thereby diminishing the production of bacteriocidal nitric oxide^25^. Furthermore, several studies have shown that feeding mosquitoes on L-Arg after *Plasmodium* infection results in a greater resistance to the parasite by enhancing nitric oxide-mediated parasite killing^24^,^27^,^28^.

**Figure 6:**
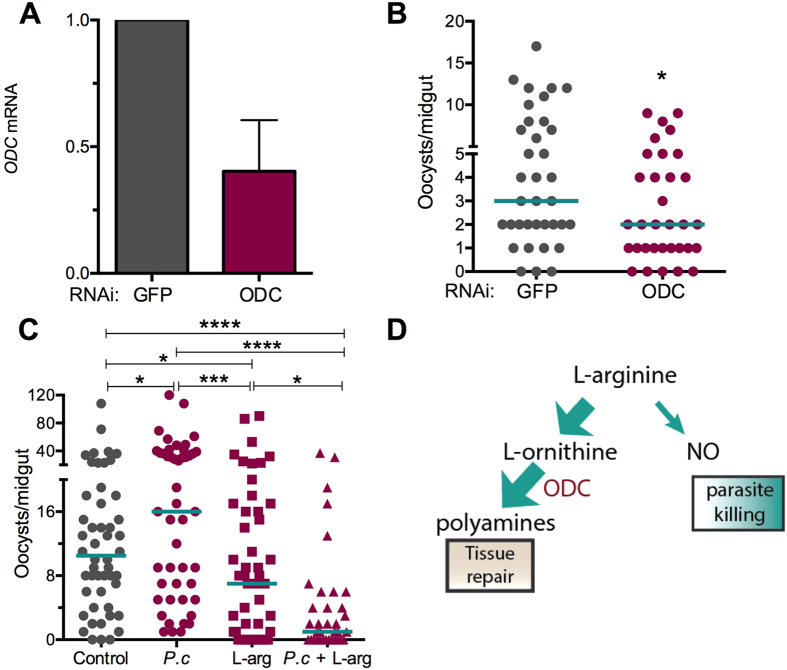
Ornithine decarboxylase (ODC) influences susceptibility to *P. falciparum* infection. (**A**) *ODC* gene silencing efficiency, as measured by qRT-PCR of mRNA abundance in whole mosquitoes; bars represent the standard error of the mean (SEM). (**B**) *P. falciparum* infection intensity of *OCD* gene-silenced mosquitoes (N = 33, *P* = 0.419) compared to GFP dsRNA-treated control mosquitoes (N = 36). (**C**) *P. falciparum* infection intensity of sugar control groups (N = 50), fungus filtrate-exposed mosquitoes (N = 47), L-Arg-fed mosquitoes (N = 50), and fungus filtrate plus L-Arg–exposed mosquitoes (N = 35). Each dot represents a single midgut, and bars represent the median infection intensity of three independent experiments, *P < 0.05, ***P < 0.001, ****P ≤ 0.0001; two-tailed Mann-Whitney test. (**D**) L-Arg is a substrate for polyamine synthesis and nitric oxide (NO) production. *Pe. chrysogenum*-secreted factors shift the balance toward polyamine synthesis, resulting in decreased parasite killing.

To determine whether ODC influences *Plasmodium* infection, we silenced the gene using dsRNA-mediated silencing after *P. falciparum*infection (Fig. 6A,B). A reduction in ODC transcript abundance by 40% (Fig. 6A) resulted in a significantly greater resistance to *Plasmodium* infection (*P* = 0.0419) in the mosquito midgut when compared to GFP dsRNA-treated controls (Fig. 6B). This result indicates an antagonistic effect of OCD on *Plasmodium* infection, which, in turn, is consistent with the use of L-Arg as its substrate. We hypothesize that an ODC over-activation results in sequestration of L-Arg, which in turn enhances parasite infection by compromising the protective response against *P. falciparum*. In order to test this hypothesis, we fed mosquitoes with the heat-inactivated fungus filtrate along with L-Arg via a sucrose solution for 48 h prior to *Plasmodium* infection (Fig. 6C). Consistent with previous studies, mosquitoes treated with L-Arg alone showed a greater resistance to parasite infection (*P* = 0.0378) than did non-L-Arg-treated controls. L-Arg supplementation negated the agonistic effect of the *Pe. chrysogenum* filtrate on parasite infection when compared mosquitoes treated with filtrate alone (*P* = 0.0001). Interestingly, co-feeding mosquitoes on both L-Arg and the fungus filtrate resulted in a greater resistance to infection (*P* = 0.0225) than did feeding on L-Arg alone. This result may reflect a synergistic effect of greater OCD activity resulting from an unlimited supply of L-Arg, perhaps leading to the production of anti-parasitic metabolites. Our results, taken together with those of previous studies, strongly suggest that *Pe. chrysogenum* influences *Plasmodium* infection through a mechanism that involves increased OCD activity and sequestration of L-Arg, which is necessary for NO-mediated *Plasmodium* killing (Fig. 6D).

## Discussion

Complex tripartite interactions between the *Anopheles* mosquito’s endogenous microbes, various mosquito physiological systems, and *Plasmodium* dictate the success of parasite infection and malaria transmission^1^,^29^. Here, we show that the presence of the natural mosquito-associated fungus, *Pe. chrysogenum*, in the *A. gambiae* midgut results in an enhanced *Plasmodium* infection of the midgut tissue. This fungus-mediated activity is exerted by a secreted heat-stable molecule(s). The fungus-secreted factor does not directly influence parasite development, independently of the mosquito, and its impact on infection is independent of the mosquito’s bacterial microbiota. The presence of *Pe. chrysogenum* in the mosquito gut also results in an increased bacterial load. The influence of the fungus-secreted molecule(s) on both parasite infection and the proliferation of the mosquito midgut bacteria suggest that it likely compromises a mosquito defense mechanism that can act against both *Plasmodium* and bacteria.

We show that ingestion of the *Pe. chrysogenum* filtrate results in the upregulation of the *ODC* gene, which encodes a rate-limiting enzyme of the polyamine biosynthesis pathway. Polyamine induction is known to interfere with NO production by NOS because of L-Arg sequestration^30^,^31^. The conversion of L-Arg into L-ornithine by ODC limits the availability of L-Arg for NO production by NOS^32^. Several studies have linked an increased L-Arg availability to greater mosquito resistance to *Plasmodium* through the action of NO, and NO is also a potent antibacterial factor; furthermore, the *NOS* gene is strongly induced by both plasmodial and bacterial infection of *A. gambiae*^24^,^33^,^34^. Chemical inhibition or RNAi-mediated depletion of OCD resulted in a greater resistance to *Plasmodium*, which is consistent with a greater availability of L-Arg. Accordingly, the effect of the *Pe. chrysogenum* filtrate on mosquito susceptibility to *Plasmodium* could be reversed by provision of L-Arg. Our data suggest that a heat-stable *Pe. chrysogenum*-secreted molecule induces the expression of OCD, which in turn causes diminished NO production by sequestering L-Arg, leading to a greater susceptibility to the *Plasmodium* parasite and midgut bacteria. Interestingly, an immunomodulatory effect of a *Chlamydia* bacterium on macrophages has been shown to be mediated by a similar mechanism involving OCD upregulation that, in turn, diminishes NO production and increases bacterial survival^35^.

These findings may have significant implications for the epidemiology and transmission of *Plasmodium* by *Anopheles* mosquitoes in the field. Like many species of *Penicillium*, *Pe. chrysogenum* is non-pathogenic to mosquitoes and may therefore not exert strong selective pressure for the development of mosquito resistance. In fact, *Penicillium* fungi have been found to be abundantly associated with field mosquitoes; also, our work suggests that mosquitoes can acquire the fungi through sugar-feeding and that the fungi will persist in the midgut tissue for up to 5 days^7^,^8^,^9^. Whether the intensity of *Pe. chrysogenum* exposure in the field is sufficient to shift the mosquito’s susceptibility to *Plasmodium* is currently unknown and also difficult to assess. *Plasmodium* infection of mosquitoes in the field is usually much lower than that achieved in the laboratory, averaging between one and three oocysts per midgut. Hence, even a relatively small increase in mosquito susceptibility to the parasite could allow mosquitoes that would not have become infected in the absence of the fungus to become infected and *Plasmodium* transmission-capable. It will be interesting to survey the midguts of field-caught mosquitoes in malaria-endemic areas for this type of fungi and assess any possible correlation with *Plasmodium* infection and epidemiological parameters.

## Methods

### Mosquito Rearing

*A. gambiae* Keele strain and wild-type *A. stephensi* Liston strain were maintained on a 10% sucrose solution with 12-h light/dark cycles at 27 °C and 80% humidity.

### Genomic DNA Extraction, PCR, and DNA Sequencing

*Pe. chrysogenum* conidia were collected and resuspended in 150 μL of sterile 1x PBS, homogenized (Bullet Blender, Next Advance) with 0.5-mm glass beads for 2 min, and the resulting homogenate incubated at 70 °C for 15 min to inactivate residual enzymes and prevent sample degradation. A phenol:chloroform extraction of nucleic acids was performed on this homogenate, and DNA yields were quantified following RNase (Qiagen) treatment. Universal fungal primers were used to amplify a region of intertranscribed spacer region of ribosomal 18 s DNA, and the PCR products were separated by electrophoresis in 1% agarose gel. The target amplicon was column-purified (Qiagen) and sent to the JHMI Genomics Core for Sanger sequencing, and the returned nucleotide sequence was searched against a non-human nucleotide database using BlastN™^14^. Table S2 lists the primers used.

### Culturing of *Pe. chrysogenum* and Collection of Conidia

*Pe. chrysogenum* was initially isolated on Yeast Peptone Dextrose Agar (YPDA) (Sigma), and subsequent cultures were maintained on Sabouraud Glucose Agar 4% (SGA) (Sigma). In brief, 4 mL of Sabouraud Dextrose Broth (SDB) was inoculated with *Pe. chrysogenum* and shaken at 30 °C for up to 72 h. These *Pe. chrysogenum* cultures were subsequently kept at 4 °C and used to inoculate SGA plates for up to 4 weeks. Using sterile technique, 100 μL of broth was added to SGA plates, and *Pe. chrysogenum* was cultured at 27 °C for 1–2 weeks or until the surface became confluent with conidia. Conidia were collected by flushing plates with 1x PBS containing 0.1% Tween 80 and by gently scraping the conidia into the solution. Glass wool filtration of this solution removed residual agar and mycelium, and the filtered conidia were centrifuged at 2,000 rpm for 10 min. The supernatant was discarded, and the conidia pellet was washed with 50 mL of sterile 1x PBS. After a second centrifugation, the pellet was resuspended in 1 mL of sterile 1x PBS and serially diluted, and a hemocytometer (Neubauer) was used to enumerate conidia by light microscopy (40x). Concentrated conidia were added to 25% glycerol for long-term storage at −80 °C in order to prepare fresh liquid starter cultures.

### Preparation of *Pe. chrysogenum* Filtrate

Four Petri dishes confluent with conidia were used for each preparation of filtrate. After washing and centrifugation as described, the PBS supernatant was discarded, and the conidia pellet was resuspended in 1 mL of sterile 10% sucrose solution. This suspension was vortexed briefly for 20 sec, and then the conidia were pelleted by centrifugation at 2,000 rpm for 10 min. After centrifugation, the supernatant was collected in a 1-mL syringe and passed through a 0.2-micron filter (Millipore). For heat inactivation, filtrates were incubated at 95 °C for 2 h. A small aliquot of filtrate was cultured on SGA plates to confirm the absence of live fungi.

### Sugar-feeding of *A. gambiae* on Conidia and Filtrate

Conidia were added to sterile 10% sucrose solution to obtain a final volume of 4 mL, and the solution was absorbed on cotton and placed in a location accessible to the mosquitoes. For filtrate preparations, approximately 1 mL of either non-treated or heat-inactivated filtrate in a sterile 10% sucrose solution was absorbed on cotton. Mosquitoes were allowed to feed on conidia or filtrate for 48 h. Conidia were administered once, whereas fresh filtrate solutions were administered on days 1 and 2. Forty-eight hours after administering conidia or filtrate, the cotton pads were removed and replaced with sterile 10% sucrose.

### *A. gambiae* Survival Assay

Three- to four-day-old adult female *A. gambiae* were allowed to feed on *Pe. chrysogenum* conidia as described above. At day 0, mosquitoes were given sterile 10% sucrose solution that was changed every 2 days. Survival of mosquitoes was monitored for 14 days, and dead mosquitoes were removed.

### Quantification of Microbiota and *Pe. chrysogenum* in the *Anopheles* Midgut

Colony forming units (CFU) from mosquito midguts were quantified in control untreated, fungi-fed, and filtrate-fed mosquitoes as described^1^,^36^. Three- to four-day-old female *A. gambiae* were fed fungi or filtrate, and then at 48 h post-feeding, the female mosquitoes were collected, surface-sterilized in ethanol, and washed with 1x PBS, and their midguts were dissected in sterile 1x PBS. Collected midguts were homogenized, and serial dilutions of homogenate were added to LB agar plates for bacterial enumeration. After incubation for 2–3 days at 27 °C under aerobic conditions, the CFUs were counted. *Pe. chrysogenum* CFU from midgut samples were similarly quantified, except that midgut samples were cultured on SGA plates containing 75 μg/mL gentamicin sulfate (Quality Biological) and 100 units/μg per mL of penicillin-streptomycin (Invitrogen). Plates were incubated for up to 4 days at 27 °C and were inspected every day for hyphal nuclei, which were enumerated to determine the fungal CFU/midgut. For the *Pe. chrysogenum* antibiotic production test, four species of mosquito-isolated bacteria (*Serratia marcences*, *Enterobacter hormaechei*, *Bacillus subtilis*, and *Staphylococcus sp.*) were grown at 30 °C on LB plates containing a disk soaked in a fungus culture filtrate solution and compared to an antibiotic-soaked disc containing 100 units/μg per mL of penicillin-streptomycin.

### Antibiotic Treatment

Adult female mosquitoes were given a sterile 10% sucrose solution containing 75 μg/mL gentamicin sulfate and 100 units/μg per mL of penicillin-streptomycin for 3 days. To validate the efficiency of the antibiotic treatment, midguts from control untreated and antibiotic-treated mosquitoes were subjected to CFU assays^22^. Where indicated, conidia or filtrate solutions were supplemented with antibiotics. Antibiotic treated cohorts were maintained on antibiotic-treated sucrose following a blood meal.

### *Plasmodium* Infection and Oocyst Enumeration

*P. falciparum* and *P. berghei* infectiuons were performed following a standard protocol^16^. For *P. falciparum* infection: At 48 h post-feeding on fungi or filtrate, mosquitoes were fed on an NF54W strain gametocyte culture mixed with human blood, through a membrane feeder at 37 °C. Engorged mosquitoes were maintained at 27 °C for up to 8 days. For *P. berghei* infection: At 2 days post-feeding on fungi or filtrate, mosquitoes were fed on Swiss Webster mice infected with the WT Anka 2.34 parasite strain. Engorged mosquitoes were maintained at 19 °C for 10 days. *P. falciparum*- and *P. berghei*-infected mosquito midguts were dissected and stained with 0.1% mercurochrome, and oocyst numbers were determined using a light microscope.

### *In vitro P. berghei* ookinete culture assays

*In vitro P. berghei* ookinete culture assays were performed as previously described using parasite infected blood from female Swiss Webster mice (6–8 weeks old) and a genetically modified *P. berghei* expressing *Renilla* luciferase under the control of an ookinete-specific promoter^37^. To assay the influence of *Pe. chrysogenum* on the ookinete culture, conidia or filtrate was added to each ookinete culture well in triplicate along with experiment-matched controls, and incubated at 19 °C for 26–28 h prior to luciferase assay he percentage inhibition of ookinete development was calculated by subtracting the blanks and expressing the luciferase units as a percentage of the control values.

### Genome-wide Microarray Analysis

Assays were conducted and analyzed as reported previously using a custom-designed full-genome 8 × 60 K *A* Agilent-based microarray platform^38^. In brief, midgut RNA from 20 mosquitoes, which had either been treated with *Pe. chrysogenum* heat-treated filtrate or left untreated, was purified using the RNeasy kit (Qiagen). Samples were labeled with the Low Input Quick Amp Labeling kit (Agilent Technologies). The array was scanned with an Agilent SureScan microarray scanner. The gene expression data were processed and analyzed as described previously^38^. Four independent biological replicate assays were performed. Self-self hybridizations have previously been used to determine the cutoff value for the significance of gene regulation to 0.75 in log_2_ scale^16^. Given the low number of regulated genes in these assays, we present genes up to a cutoff value of 0.50 in log_2_ scale, which corresponds to 1.40-fold regulation. Numeric gene expression data are presented in Table 1.

### dsRNA-mediated Gene Silencing

*The Odc* gene was depleted from adult female mosquitoes using established RNAi methodology^16^. RNAi assays were repeated three times, using *GFP* dsRNA as a control. Gene silencing was verified at 3 days post-injection using RNA extracted from five whole mosquitoes per biological replicate, and two technical replicates by real-time RT-PCR with Sybr Green PCR Master Mix (Applied Biosystems) using the ABI StepOnePlus Real-Time PCR System and ABI StepOne Software. The ribosomal protein S7 gene was used to standardize and verify ODC silencing. The primers used to produce PCR Amplicons for dsRNA synthesis and qRT-PCR are given in Table S2.

### L-Arginine Treatment

Mosquitoes were treated for 48 h prior to *P. falciparum* infection with either *Pe. chrysogenum* heat-inactivated filtrate, 0.2% L-arginine (L-Arg), or both, all in 10% sucrose solution. After *P. falciparum* infection, the mosquitoes were maintained with sugar throughout the 8 days of infection incubation, or with 0.2% L-Arg-supplemented sugar in the case of the groups treated previously with L-Arg.

### Statistical Analysis

The Graphpad Prism 6 (Graphpad Prism^®^) software package was used to perform statistical analyses. The particular test used is indicated in the legend of each respective figure. See Table S1 for a summary of the statistics.

## Additional Information

**How to cite this article**: Angleró-Rodríguez, Y. I. *et al*. A natural *Anopheles*-associated *Penicillium chrysogenum* enhances mosquito susceptibility to *Plasmodium* infection. *Sci. Rep.* **6**, 34084; doi: 10.1038/srep34084 (2016).

## Supplementary Material

Supplementary Information

## Figures and Tables

**Table 1:** Regulated genes 48 h after heat-inactivated *Pe. chrysogenum* filtrate treatment.

Gene ID	Gene description	Functional Group	Log_2_
AGAP011806	ornithine decarboxylase	M	1.555
AGAP011302	alkaline phosphatase	D	1.499
AGAP000535	hypothetical protein	D	1.495
AGAP008212	cytochrome P450 (CYP6M2)	R/S	1.465
AGAP000376	transferrin	I	1.092
AGAP001198	chymotrypsin	DIG	1.062
AGAP008293	trypsin (TRY7)	DIG	0.992
AGAP009563	myotubularin	D	0.842
AGAP004916	hypothetical protein	I	0.830
AGAP000586	hypothetical protein	UNK	0.822
AGAP002865	cytochrome P450 (CYP6P3)	R/S	0.809
AGAP003934	battenin	D	0.797
AGAP006385	trypsin-like	DIG	0.777
AGAP008100	spire	D	0.752
AGAP000647	hypothetical protein	D	0.644
AGAP005241	hypothetical protein	UNK	0.639
AGAP002867	cytochrome P450 (CYP6P4)	R/S	0.599
AGAP007990	glucosyl/glucuronosyl transferases	M	0.589
AGAP008982	cation-efflux system protein	TRP	0.558
AGAP003209	C-4 methylsterol oxidase	M	0.548
AGAP008294	trypsin 3	DIG	0.530
AGAP004918	fibrinogen	I	0.513
AGAP000561	kinesin family member 5	C/S	0.512
AGAP001652	Lipase 3	M	0.511
AGAP009924	hypothetical protein	UNK	0.507
AGAP002852	Niemann-pick type C-like	I	−0.716
AGAP004484	Fe/S domain-containing protein	UNK	−0.671
AGAP009212	serpin 6	I	−0.667
AGAP008688	hypothetical protein	UNK	−0.640
AGAP003350	P-enolpyruvate carboxykinase	M	−0.565
AGAP007621	cytochrome c oxidase VIIc	R/S	−0.558

(M) metabolism, (D) diverse, (R/S) redox/stress, (I) immunity, (DIG) digestion, (C/S), cytoskeletal and structural, (TRP), transport, (UNK) unknown.
